# Analysis of reactive aldehydes in urine and plasma of type-2 diabetes mellitus patients through liquid chromatography-mass spectrometry: Reactive aldehydes as potential markers of diabetic nephropathy

**DOI:** 10.3389/fnut.2022.997015

**Published:** 2023-01-16

**Authors:** Carla Harkin, Diego Cobice, Joanne Watt, Mary Jo Kurth, Simon Brockbank, Stephanie Bolton, Frances Johnston, Anna Strzelecka, John V. Lamont, Tara Moore, Peter Fitzgerald, Mark W. Ruddock

**Affiliations:** ^1^Biomedical Sciences Research Institute, Ulster University, Coleraine, United Kingdom; ^2^Clinical Studies Group, Randox Laboratories Ltd., Randox Science Park, Antrim, United Kingdom; ^3^Renal Unit, Antrim Area Hospital, Northern Health and Social Care Trust, Antrim, United Kingdom; ^4^Diabetic Services, Whiteabbey Hospital, Northern Health and Social Care Trust, Newtownabbey, United Kingdom

**Keywords:** type-2 diabetes, diabetic nephropathy, 4-hydroxynonenal (4-HNE), 4-oxo-2-nonenal (4-ONE), 4-hydroxyhexenal (4-HHE), pentanal, methylglyoxal, glyoxal

## Abstract

**Introduction:**

Diabetes is a major public health issue that is approaching epidemic proportions globally. Diabetes mortality is increasing in all ethnic groups, irrespective of socio-economic class. Obesity is often seen as the main contributor to an increasing prevalence of diabetes. Oxidative stress has been shown to trigger obesity by stimulating the deposition of white adipose tissue. In this study, we measured reactive aldehydes by liquid chromatography-mass spectrometry (LC-MS), in the urine and plasma of type-2 diabetic mellitus (T2DM) patients, as potential surrogates of oxidative stress. Our hypothesis was that reactive aldehydes play a significant role in the pathophysiology of diabetes, and these reactive species, may present potential drug targets for patient treatment.

**Materials and methods:**

Study participants [*N* = 86; control *n* = 26; T2DM *n* = 32, and diabetic nephropathy (DN) *n* = 28] were recruited between 2019 and 2020. Urine and blood samples were collected from all participants, including a detailed clinical history, to include patient behaviours, medications, and co-morbidities. Reactive aldehyde concentrations in urine and plasma were measured using pre-column derivatisation and LC-MS, for control, T2DM and DN patients.

**Results:**

Reactive aldehydes were measured in the urine and plasma of control subjects and patients with T2DM and DN. In all cases, the reactive aldehydes under investigation; 4-HNE, 4-ONE, 4-HHE, pentanal, methylglyoxal, and glyoxal, were significantly elevated in the urine and serum of the patients with T2DM and DN, compared to controls (*p* < 0.001) (Kruskal–Wallis). Urine and serum reactive aldehydes were significantly correlated (≥0.7) (*p* < 0.001) (Spearman rho). The concentrations of the reactive aldehydes were significantly higher in plasma samples, when compared to urine, suggesting that plasma is the optimal matrix for screening T2DM and DN patients for oxidative stress.

**Conclusion:**

Reactive aldehydes are elevated in the urine and plasma of T2DM and DN patients. Reactive aldehydes have been implicated in the pathobiology of T2DM. Therefore, if reactive aldehydes are surrogates of oxidative stress, these reactive aldehyde species could be therapeutic targets for potential drug development.

## Introduction

Diabetes is a chronic condition that arises from an inability to either produce or utilise insulin to maintain glucose homeostasis (1). Diabetes and its associated multisystem complications have severe implications for global health (2). The International Diabetes Federation has published data indicating that the prevalence of diabetes in adults aged 20–79 in 2019 was 463 million people (9.3% of the global population) and is projected to increase to 578 million by 2030 (3). Therefore, diabetes represents a serious threat to global health (4). Diabetic complications in 2019 were associated with 4.2 million deaths (5). Furthermore, it is estimated that 50.1% of the global population with diabetes, currently remain undiagnosed. The subsequent costs of managing diabetic patients in 2017 exceeded $760 billion (6).

Diabetes is classified either Type 1 (T1DM) or Type 2 (T2DM). However, other classifications include, diabetes in pregnancy (DIP) and gestational diabetes (GDM) (7). Diabetes can arise from pre-existing conditions, such as, endocrine disorders, viral infection, and genetic disorders (e.g., Prader-Willi syndrome, Down’s syndrome, and Friedreich’s ataxia) (8).

Type-2 diabetic mellitus is the most common type of diabetes and accounts for almost 90% of all cases worldwide. Diagnosis is generally made in adulthood, however, the incidence in children and young adults is increasing due to sedentary lifestyles and high energy dietary practices (behavioural) (9).

The diabetic environment is one of oxidative stress and inflammation, causing damage to renal cells and loss of kidney function e.g., diabetic nephropathy (DN) (10). DN is a leading cause of end stage renal disease (ESRD) (11), accounting for almost 28% of all patients requiring dialysis or renal replacement therapy in the UK (12).

The economic impact of late-stage DN is significantly higher than that of other microvascular complications. It has been estimated that medical costs for T2DM patients with clinical nephropathy ($9,700) are over 3-times higher than that of patients without nephropathy or microalbuminuria ($3,000). In patients where DN progresses to ESRD requiring dialysis, costs have been estimated at $41,117 (13–15).

Screening high-risk patients for chronic kidney disease (CKD) e.g., diabetics, is cost-effective, according to the World Health Organisation (16). Risk factors for DN progression in patients with T2DM include; age, body mass index (BMI), smoking status, glycated haemoglobin (HbA1c), systolic blood pressure (hypertension), HDL cholesterol, triglyceride, urine albumin: creatinine ratio, and estimated glomerular filtration rate (eGFR) (17, 18).

Diabetic nephropathy is characterised by a variety of structural and molecular changes within the nephron. Chronic inflammation (19), pro-fibrotic milieu (20), and glomerulosclerosis result in pathological changes and potential loss of nephron activity (21). Nephrons are multifunctional filtration systems which are in the cortex, which, together with capillaries and collecting ducts, regulate waste and electrolyte homeostasis. Structurally, they are composed of a convoluted capillary that feeds into the surrounding Bowman’s capsule, delivering filtrate to the epithelial tubule and collecting ducts. Glomerular capillaries are surrounded by specialised cells, podocytes, which envelope the vessels attached by mesangial cells that are in the space between the capillaries. Fenestrated endothelium, podocytes, and the glomerular basement membrane (GBM) formed by cellular secretions constitutes the glomerular filtration barrier (GFB) through which acellular filtrate passes (water, small solutes, and low molecular weight proteins). Mesangial cells contribute to the generation of extracellular matrix (ECM) and control the availability of the surface area for filtration by expansion and contraction mechanisms, regulated by systemic neural input and local neural input (macula densa). This structure is an area of closely packed cells which line the wall of the distal tubule where the thick ascending limb of the Loop of Henle meets the distal convoluted tubule, proximate to the glomerulus.

Diagnosis of DN involves assessment of urinary protein (proteinuria/albuminuria), eGFR and clinical features, such as those described earlier (22). However, these tests have limitations, and there is a need for novel diagnostic biomarkers to monitor progression. Recent studies have suggested that the currently methodology, Modification of Diet in Renal Disease (MDRD) and Chronic Kidney Disease Epidemiology Collaboration (CKD-EPI) (23), potentially underestimate eGFR in T2DM patients, when compared to measurements based on clearance of the tracer, chromium-51 (24).

Oxidative stress is a common pathogenic factor that is thought to lead to insulin resistance, β-cell dysfunction, impaired glucose tolerance, and impaired fasting glycaemia (25). Products of lipid peroxidation have received considerable attention, as they serve as indices for oxidative stress, given that lipids are susceptible to oxidation *in vivo* (26). As a result, various lipid products e.g., reactive aldehydes, have been evaluated, using diverse methods and techniques e.g., Liquid Chromatography–Mass Spectrometry (LC-MS).

Reactive oxygen species (ROS), such as superoxide anion, hydrogen peroxide, and hydroxyl radicals, are generated by several enzymatic and non-enzymatic reaction pathways (27). Overproduction and insufficient removal of ROS induces oxidative stress, which has been linked to heart failure, endothelial dysfunction, and acute tubular necrosis (ATN), the latter being a reversible loss of renal function incurred from ischemic or nephrotoxic insults (28). Such insults instigate a cascade of processes; hemodynamic alterations, aberrant vascular responses, sub-lethal and lethal cell damage, inflammatory responses, and nephron obstruction, that initiate and maintain ATN. Recent data indicate that oxidative stress may trigger obesity, insulin resistance, T2DM and ATN, or at least contribute to disease progression (29–32).

**FIGURE 1:**
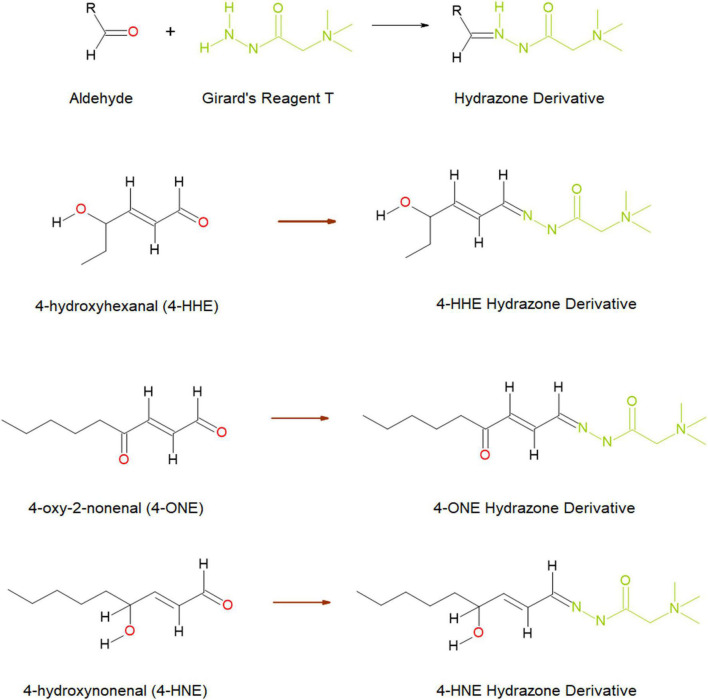
Schematic diagram of the reaction between aldehydes and Girard’s reagent T forming hydrazone derivatives **(top)**, and the hydrazone formed through reaction with three reactive aldehydes; 4-hydroxyhexenal (4-HHE), 4-oxo-2-nonenal (4-ONE), and 4-hydroxynonenal (4-HNE) **(below)**. Image taken from Harkin, et al. (33) and reproduced under the Creative Commons License (http://creativecommons.org/licenses/by/4.0/).

In this study, we investigated whether the reactive aldehydes (4-HNE, 4-ONE, 4-HHE, pentanal, methylglyoxal, and glyoxal) (Figure 1) were elevated in the urine and plasma of patients diagnosed with T2DM and/or DN. Our hypothesis was that if ROS is elevated in patients with obesity and obesity is a causative factor implicated in the pathophysiology of T2DM, and/or progression to DN, then the levels of these reactive aldehydes in both the urine and plasma of these patients may act as markers, or indicators, of disease severity.

## Materials and methods

### Chemicals and reagents

Acetonitrile (ACN) (≥99.9% v/v) and methyl tert-butyl ether (MTBE), (∼99% v/v) were obtained from Fisher Scientific, Loughborough, Leicestershire, UK. Methanol (MeOH) (≥99.9% v/v), trifluoroacetic acid (TFA, 99% v/v) and formic acid (∼98% v/v) were HPLC grade and obtained from Sigma-Aldrich, Gillingham, Dorset, UK. MilliQ water was produced in house by an Elga water purifier (∼18 Ohm). Dinitrophenylhydrazine HCl (DNPH) was purchased from Sigma-Aldrich, Gillingham, Dorset, UK. Reactive aldehyde standard solutions glyoxal and methylglyoxal (both 40% v/v in water), *p*-anisaldehyde (98% v/v) and pentanal (valeraldehyde) (≥97.5% v/v) were obtained from Sigma-Aldrich, Gillingham, Dorset, UK. Remaining aldehyde standards, 4-hydroxyhexenal (4-HHE) (≥98.5% v/v), 4-hydroxynonenal (4-HNE) (≥99.5% v/v) and 4-oxo-2-nonenal (4-ONE) (≥99.0% v/v) were obtained from Cambridge Bioscience, Cambridge, UK. Working stock solutions (1 mg/ml) of aldehyde standards were prepared in ACN and stored at −20°C. until analysis. The solution was stable for 1 week at −20°C.

### Liquid chromatography–mass spectrometry (LC-MS)

Participant blood and urine samples were tested for relative concentrations of reactive aldehyde concentrations. For each participant plasma sample, 50 μl was spiked with an internal standard (ISTD) (*p*-anisaldehyde, 0.020 ng/ml) and 500 μl of −20°C ACN containing 0.1% v/v formic acid. Samples were vortexed for 30 s and then centrifuged at 13,000 rpm for 5 min to remove precipitated protein. The supernatant was transferred to a clean 1.5 ml tube and 700 μl of methyl tert-butyl ether (MTBE) was added before mixing and centrifugation (13,000 rpm for 5 min). The upper layer (1 ml) was evaporated at room temperature (RT) under nitrogen gas. DNPH (100 μl, 0.5 mg/ml in ACN) was added and the samples were left at RT for 2 h. The reaction was quenched with 100 μl water (MilliQ) and transferred to a 1.5 ml high-performance liquid chromatography (HPLC) amber vial with a 250 μl insert. Samples were analysed immediately.

Each participant urine sample (200 μl) was diluted with water 1:1 v/v and desalted using ZipTip^®^ (Millipore, Merck, Gillingham, Dorset, UK). The sample was spiked with an internal standard (*p*-anisaldehyde 0.025 ng/ml). Samples were evaporated to dryness and reacted with the derivatisation reagent DNPH, as detailed above. For reactive aldehyde analysis, an Agilent 1250 ultra HPLC system (Agilent Technologies Inc., Santa Clara, CA, US) coupled to a 6500 Qtrap (ABSciex, LLC, MA, US) was used. Samples and standards were separated on a Luna extended, reverse phase C_18_ column, dimensions, 50 × 2.1 mm with 3.0 μm particle size (Phenomenex Inc., Torrance, CA, US) at 40°C, in gradient mode using mobile phases (A) 100% v/v ACN and (B) 60:40 v/v water:ACN at a flow rate of 0.40 ml/min (Table 1) achieving a total run time of 35.1 min. Mobile phases were degassed by ultrasonication for 15 min prior to analysis. The autosampler was kept at 5°C and injection volume was 20 μl. LC-MS chromatograph-spectrum of the determined compounds have been previous published (33).

**TABLE 1:** Liquid chromatography (LC) gradient profile.

Time (min)	% A	% B
0.0	0	100
5.0	0	100
25.0	100	0
30.0	100	0
30.1	0	100
35.0	0	100

Mass spectrometer (MS) parameters: turbo ion spray source in negative mode, temperature 550°C, curtain gas: 35 Arb units, GSI and GSII: 32 Arb units, Ion spray voltage −4500 V. Reactive aldehydes were detected using multiple reaction monitoring (MRM) mode (Table 2).

**TABLE 2:** *m/z* transition and source parameters.

Reactive aldehyde	Parent (*m/z*)	Product (*m/z*)	CE (V)	DP (V)	CXP (V)	EP (V)
4-HNE	335.1	163.1	33	68	10	11
4-ONE	333.1	161.1	35	98	10	10
4-HHE	293.1	170.0	41	80	10	9
Pentanal	252.2	162.1	35	82	10	10
Methylglyoxal	251.2	172.2	35	85	10	5
Glyoxal	417.2	172.2	33	90	10	8
**ISTD**	315.1	**152.1**	**32**	**80**	**10**	**10**

CE, collision energy; DP, de-clustering potential; CXP, collision exit potential; EP, entrance potential; ISTD, internal standard. Internal standard (ISTD) values are indicated in bold.

Sample diluents and blank samples were injected in duplicate to condition the column at the beginning of the run and inspected for interfering peaks. Calibration standards were then injected in duplicate followed by two further blank injections. Samples were analysed in duplicate in batches of 10 samples with sample diluents and controls in duplicate between each run. All samples were analysed in duplicate and linear regression analysis for all standards [ratio against internal standard (ISTD)] was used to calculate the content of reactive aldehyde in the samples using an y = ax + b equation. The methodology employed for the LC-MS is as previously described (33). Data were reported as the average of the two injections. The method was fully validated as per International Council for Harmonisation of Technical Requirements for Pharmaceuticals for Human Use guidelines on bioanalytical method validation (Supplementary Data Sheet 1; 33).

### Study participants

Study participants [*N* = 86; control *n* = 26 (30.2%); T2DM *n* = 32 (37.2%), and DN *n* = 28 (32.6%)] were recruited between 2019 and 2020 from the Diabetic Clinic, Whiteabbey Hospital, Northern Health and Social Care Trust, Newtownabbey, Northern Ireland, UK; Renal Unit, Antrim Area Hospital, Northern Health and Social Care Trust, Antrim, Northern Ireland, UK; and the University of the Third Age (U3a), Belfast, UK.

Study participants were asked to complete a comprehensive questionnaire detailing their medical history, lifestyle, and behaviours (alcohol consumption and smoking habits), current medications and comorbidities. Blood pressure (mmHg) readings were recorded following completion of the patient questionnaire. Participants’ height (cm), weight (kg), estimated glomerular filtration (eGFR) rate, and HbA1c were also recorded.

Patients <18 years of age with autoimmune disease or who had a condition or illness that impacted kidney function, were excluded from the study. Formal written consent was obtained from all study participants. Urine and blood samples were collected in an outpatient setting. Patient sample collection has been detailed previously (34). Briefly, venous blood (10 ml) and urine (10 ml) (where available) was collected from DN participants. Venous blood (20 ml) and urine (10 ml) samples were obtained from all other study participants.

All participants received detailed information on the study and were also invited to ask questions. The study was approved by HSC Chelsea/London Ethics Approval Committee. Research governance permission was granted by Northern Health and Social Care Trust. The study was conducted according to Standards for Reporting Diagnostic Accuracy (STARD) guidelines to facilitate interstudy comparison (35).

### Statistical analysis

Statistical analysis was undertaken using R (36). Urine and plasma reactive aldehyde data were analysed using Kruskal–Wallis (KW) to identify which reactive aldehydes were differentially expressed between control, T2DM and DN groups. Statistical significance was taken at the *p* < 0.05 level and results are presented as mean ± SD, where appropriate. Spearman’s rho correlations were also performed. Correlations ≥0.7 were considered significant. Stars of significance *p < 0.05, ^**^p < 0.01, ^***^p < 0.001, and ^****^p < 0.0001. Area under the receiver operator characteristic (AUROC) were also calculated for each reactive aldehyde (urine and plasma) comparing control to T2DM, control to DN, and T2DM to DN groups.

## Results

### Clinical and demographic characteristics of the study participants

Clinical and demographic characteristics of the study participants are described in Supplementary Table 1. Dip stick urinalysis, Bradford assay, creatinine, osmolality, eGFR, HbA1c, urine, and serum biomarkers results e.g., total antioxidant status (TAS), have been described previously (34).

### Reactive aldehyde results

#### Detection of reactive aldehydes in urine

**FIGURE 2:**
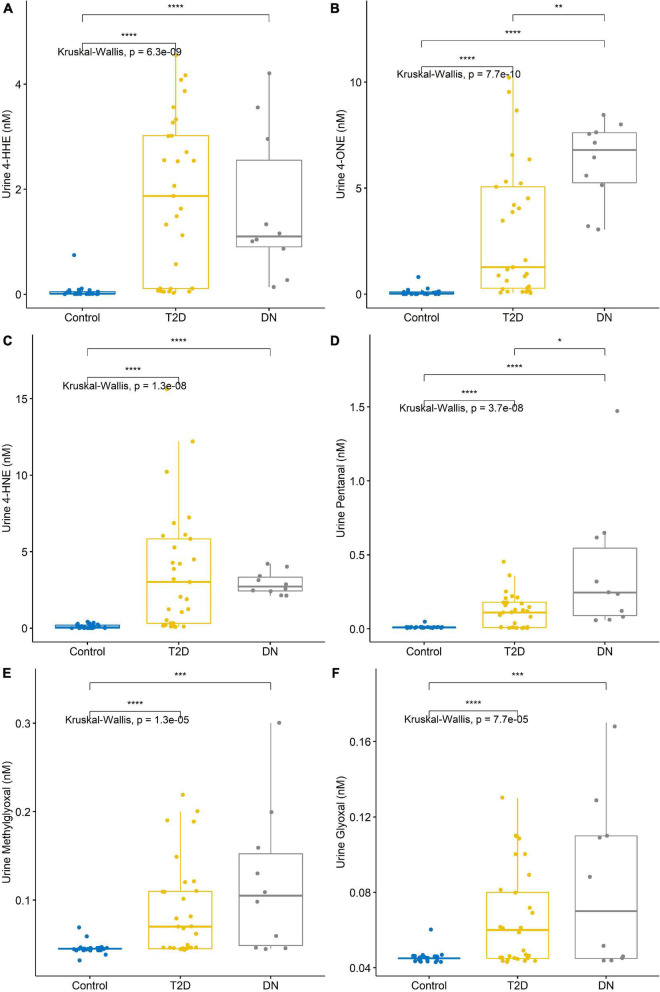
Reactive aldehyde urine levels across control, type-2 diabetic mellitus (T2DM) and diabetic nephropathy (DN) groups **(A)** 4-hydroxynonenal (4-HHE), **(B)** 4-oxo-2-nonenal (4-ONE), **(C)** 4-hydroxyhexenal (4-HNE), **(D)** pentanal, **(E)** methylglyoxal, **(F)** glyoxal. Stars of significance *p < 0.05, **p < 0.01, ***p < 0.001, and ****p < 0.0001.

The detection of reactive aldehydes in participant urine: alterations in reactive aldehyde concentrations between participant groups were investigated using DNPH pre-derivatisation and LC-MS. 4-ONE and pentanal concentrations were found to be statistically significant across control vs. T2DM, T2DM vs. DN, and control vs. DN (Table 3 and Figure 2). The remaining aldehydes were significantly different between (a) control vs. T2DM groups and (b) control vs. DN groups. All reactive aldehydes followed a linear increasing trend from the control > T2DM > DN groups, except for two reactive aldehydes, 4-HNE and 4-HHE. Based on our results, urine 4-ONE and urine pentanal represented the most effective reactive aldehydes for differentiating T2DM from DN patients.

**TABLE 3:** Reactive aldehydes in urine (mean ± SD).

Reactive aldehyde (Urine)	Control (*n* = 26) (nM)	T2DM (*n* = 29) (nM)	DN (*n* = 10) (nM)	*P*-value (KW)
4-HNE	0.111 ± 0.125	3.723 ± 3.939	2.939 ± 0.743	<0.001
4-ONE	0.088 ± 0.163	2.962 ± 3.110	6.222 ± 1.929	<0.001
4-HHE	0.058 ± 0.145	1.858 ± 1.523	1.655 ± 1.408	<0.001
Pentanal	0.011 ± 0.008	0.119 ± 0.112	0.387 ± 0.437	<0.001
Methylglyoxal	0.046 ± 0.007	0.089 ± 0.054	0.119 ± 0.083	<0.001
Glyoxal	0.046 ± 0.003	0.066 ± 0.026	0.084 ± 0.045	<0.001

**FIGURE 3:**
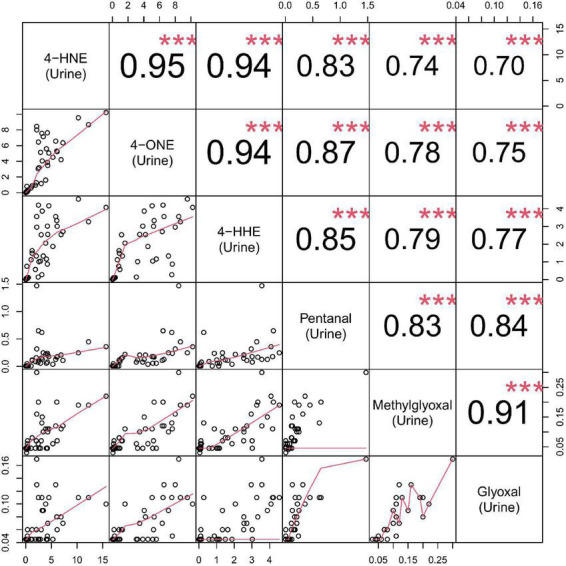
Correlation matrix chart for urine reactive aldehydes. **Top right**–the (absolute) value of the correlation (R) using Spearman’s rho with stars of significance. **Bottom left**–the bivariate scatterplots, with black circles indicating points and red fitted line. Correlations ≥0.7 were considered significant. Stars of significance ***p < 0.001.

A correlation matrix is shown in Figure 3. Reactive aldehydes, in urine, were significantly elevated in T2DM and DN patients, with respect to control patients. AUROC for reactive aldehydes under investigation in urine are described in Table 4.

**TABLE 4:** Area under the receiver operator characteristic comparing urine reactive aldehydes: Control vs. type-2 diabetic mellitus (T2DM), control vs. diabetic nephropathy (DN), and T2DM vs. DN.

Reactive aldehyde	AUROC		
	Control vs. T2DM	Control vs. DN	T2DM vs. DN
4-HNE	0.999	0.992	0.801
4-ONE	0.980	1.000	0.656
4-HHE	0.988	1.000	0.535
Pentanal	0.869	0.997	0.731
Methylglyoxal	0.776	0.907	0.757
Glyoxal	0.671	0.891	0.721

#### Detection of reactive aldehydes in plasma

**FIGURE 4:**
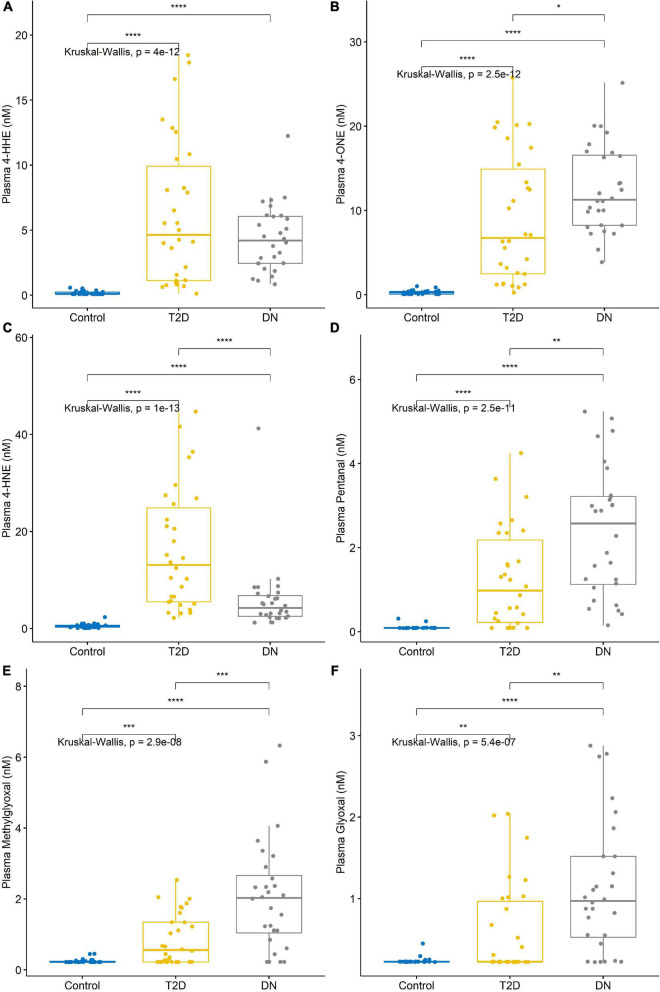
Reactive aldehyde plasma levels across control, type-2 diabetic mellitus (T2DM) and diabetic nephropathy (DN) groups **(A)** 4-hydroxynonenal (4-HHE), **(B)** 4-oxo-2-nonenal (4-ONE), **(C)** 4-hydroxyhexenal (4-HNE), **(D)** pentanal, **(E)** methylglyoxal, **(F)** glyoxal. Stars of significance *p < 0.05, **p < 0.01, ***p < 0.001, and ****p < 0.0001.

The detection of reactive aldehydes in plasma: Alterations in plasma reactive aldehyde concentrations between participant groups were investigated using DNPH pre-derivatisation and LC-MS. A similar pattern of reactive aldehydes across groups to that shown in the urine, was also observed in the plasma (Table 5 and Figure 4). Albeit the levels of reactive aldehydes in the plasma were generally higher than that observed in the urine. To evaluate the potential of reactive aldehydes as surrogates of oxidative stress, we compared the plasma results for the aldehydes with TAS. The AUROC for TAS to differentiate control vs. T2DM was 0.792, and for T2DM vs. DN was 0.545 (34). In contrast, the plasma aldehyde 4-ONE had an AUROC of 0.938 and 0.803 for control vs. T2DM, and T2DM vs. DN, respectively.

**TABLE 5:** Reactive aldehydes in plasma (mean ± SD).

Reactive aldehyde (Plasma)	Control (*n* = 26) (nM)	T2DM (*n* = 30) (nM)	DN (*n* = 28) (nM)	*P*-value (KW)
4-HNE	0.564 ± 0.467	16.158 ± 12.458	5.909 ± 7.387	<0.001
4-ONE	0.299 ± 0.248	9.126 ± 7.618	12.486 ± 5.125	<0.001
4-HHE	0.178 ± 0.146	6.237 ± 5.589	4.369 ± 2.568	<0.001
Pentanal	0.105 ± 0.052	1.252 ± 1.193	2.396 ± 1.538	<0.001
Methylglyoxal	0.252 ± 0.062	0.860 ± 0.715	2.078 ± 1.572	<0.001
Glyoxal	0.238 ± 0.045	0.599 ± 0.569	1.126 ± 0.811	<0.001

**FIGURE 5:**
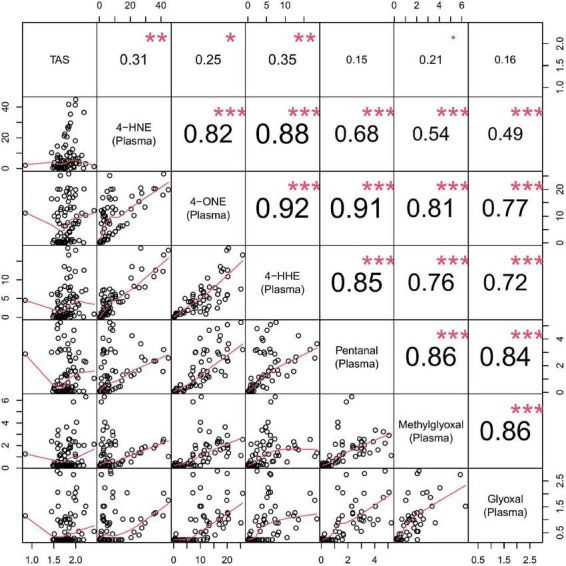
Correlation matrix chart for plasma reactive aldehydes. **Top right**–the (absolute) value of the correlation (R) using Spearman’s rho with stars of significance. **Bottom left**–the bivariate scatterplots, with black circles indicating points and red fitted line. Correlations ≥0.7 were considered significant. Stars of significance ***p < 0.001.

A correlation matrix is shown in Figure 5. Reactive aldehydes, in plasma, were significantly elevated in T2DM and DN patients, with respect to control patients. AUROC for reactive aldehydes under investigation in plasma are described in Table 6. There was no significant correlation (≥0.7) of the reactive aldehydes with TAS.

**TABLE 6:** Area under the receiver operator characteristic comparing plasma reactive aldehydes: Control vs. type-2 diabetic mellitus (T2DM), control vs. diabetic nephropathy (DN), and T2DM vs. DN.

Reactive aldehyde	AUROC		
	Control vs. T2DM	Control vs. DN	T2DM vs. DN
4-HNE	0.922	1.000	0.486
4-ONE	0.938	1.000	0.803
4-HHE	0.934	0.992	0.507
Pentanal	0.820	1.000	0.743
Methylglyoxal	0.804	0.844	0.598
Glyoxal	0.763	0.788	0.595

## Discussion

There are different types of nutritionally mediated oxidative stress sources that trigger inflammation. Much information indicates that high intake of macronutrients can promote reactive aldehyde formation that subsequently contribute to inflammation *via* nuclear factor-kappa B-mediated cell signalling pathways. Dietary carbohydrates, animal-based proteins, and fats are important because they may contribute to the long-term consequences of nutritionally mediated inflammation. Reactive-aldehydes *via* oxidative stress is a central player of metabolic ailments (such as T2D) associated with high-carbohydrate and animal-based protein diets and excessive fat consumption. However, the molecular mechanisms of nutritionally mediated oxidative stress are complex and poorly understood. Taken together, a better understanding of the role of reactive aldehydes as by-product of oxidative stress in inflammation-mediated diseases such as metabolic syndrome and T2DM would provide a useful approach. This is because reactive aldehydes *via* oxidative stress can be mediated by both extrinsic and intrinsic factors, hence providing a plausible means for the prevention of metabolic disorders using antioxidant-based nutritional/nutraceutical approaches.

Oxidative stress is caused by an accumulation of ROS in cells and tissues (37). Increased oxidative stress has been linked directly to weight gain (38). Obesity augments oxidative stress (39). Therefore, there is a complicated relationship between cause and effect in obese patients. Furthermore, there is a positive association between weight gain and inflammatory response (40). Moreover, obesity has been shown to be the principle causative factor in the development of metabolic syndrome (41). Therefore, it is somewhat unsurprising, that metabolic syndrome is often characterised by oxidative stress.

Lipid peroxidation, the oxidative degradation of lipid membranes, generates >200 types of aldehydes, many of which are highly reactive and toxic to the body (42). The cytotoxic products of lipid peroxidation can cause covalent modification of macromolecules, which cause long-lasting and significant biological consequences (43) e.g., changes in fluidity and permeability, modulation of ion transport, and inhibition of metabolic processes (44).

Factors that increase the risk of oxidative stress include, diets that are high in fat, increased sugar consumption, highly processed foods, exposure to radiation, tobacco products, alcohol consumption, certain medications, environmental pollution, excessive exercise, and occupational exposure to harmful chemicals.

Vitamin E (α-tocopherol) and C (ascorbic acid) supplementation have been shown to have a protective effect by modulating or preventing damage induced by oxidative stress (45). Vitamin E is recognised as nature’s most effective, lipid soluble, chain-breaking antioxidant, that prevents cellular membranes from being attacked by lipid peroxyl radicals. These antioxidants inhibit the chain reaction of free radical formation.

Under optimal physiological conditions, cells control ROS levels by modulating their generation. To protect the body from free radical toxicity, cellular antioxidant defence mechanisms exist to modulate the production of ROS (46).

ROS damage DNA through strand breaks (genomic instability and mutations) and base oxidation (47). If the DNA remains unrepaired, cells can undergo apoptosis and/or oncosis. Therefore, increased ROS levels have an important role in the initiation and pathophysiology of carcinogenesis.

Previous studies have suggested that reactive aldehydes may contribute to DN (48–50). Furthermore, increased reactive aldehydes levels have been found in the retinas of human post-mortem diabetic donors (51). The imbalance between the production of ROS and the body’s antioxidant defence system and response, show a direct link to the pathobiology of diabetes and its associated complications. In patients with already compromised immune systems, the production of ROS, and the body’s inability to neutralise or defend against oxidative stress, can result in accelerated atherosclerosis and vascular pathologies.

Aldehydes in biological matrices are known for their volatility, polarity, and biochemical instability making their measurement challenging. For this reason, derivatisation is commonly used to analyse low molecular weight aldehydes in complex matrices. MS provides increased selectivity, specificity and sensitivity over that achievable with fluorescence detection (52, 53).

The objective of our study was to identify if reactive aldehydes (in both urine and plasma), could be used to differentiate and predict progression of DN in patients with T2DM and DN using a validated LC-MS analytical assay.

Unsurprisingly, the level of reactive aldehydes under investigation was significantly elevated in both the urine and plasma of patients diagnosed with T2DM and DN. Moreover, the concentration of the reactive aldehydes was almost 6-fold higher in the plasma compared to urine. Furthermore, the reactive aldehydes were diagnostic, in that they were able to differentiate between patients with T2DM and/or DN, from control participants when compared to TAS.

Reactive aldehyde-conjugating therapies exist for cardiovascular disease (54). However, technical challenges have limited their use clinically, with many compounds failing clinical trials due to unforeseen safety concerns. However, it is reasonable to assume that patients taking high-dose antioxidants, which are safe, could reduce their urine and plasma reactive aldehydes. The question remains that if there is a correlation between reactive aldehydes, either in the urine or plasma, and if this correction is related to disease severity. Thus, if reactive aldehydes were monitored over time, as a function of antioxidant status, and there is attenuation of oxidative stress, then further longitudinal studies are warranted to investigate which antioxidant produces the greatest effect (e.g., Vitamin C, Vitamin E and/or glutathione, or combination(s) thereof).

## Conclusion

Reactive aldehydes have been implicated in the pathophysiology of diabetes. Oxidative stress plays a pivotal role in the development of diabetic complications, including microvascular and cardiovascular disease. Dyslipidemia in diabetic patients induces microangiopathies that augment oxidative stress leading to atherosclerosis.

In this pilot study, we have demonstrated that reactive aldehydes are elevated in the urine and plasma of T2DM and DN patients, with respect to control participants. Using reactive aldehydes as surrogates of oxidative stress, we suggest that LC-MS could be employed in the diabetic clinic to monitor oxidative damage induced by reactive aldehydes and that these reactive species may offer potential drug targets for therapeutic intervention e.g., antioxidant therapy. Moreover, reactive aldehydes constitute a protentional diagnostic biomarker for diabetes and DN and not only/not mainly a therapeutic target. Furthermore, if there is a direct correlation between reactive aldehyde levels and disease severity, measuring one, or more, of these reactive aldehydes may indicate if treatment management is effective. LC-MS is commonly deployed in hospitals e.g., vitamin D measurement (55). However, current costs to measure reactive aldehydes by LC-MS may be prohibitive. Albeit, as technology develops, LC-MS may become much more routine and cost effective.

Future studies will investigate whether reactive aldehyde levels can be attenuated using antioxidants and/or medications, or combinations thereof. Moreover, do we observe a decrease in patient symptomology using antioxidant therapy i.e., reduced insulin resistance and improved glucose homeostasis? These questions will be addressed in future longitudinal studies.

Commonly employed methods to test if a biomarker, or in this case, reactive aldehyde, will add to risk prediction models are normally based on (i) model discrimination, (ii) model calibration, and (iii) risk reclassification. Therefore, addition of the reactive aldehydes, to known risks of T2DM and DN, would allow clinicians to both identify patients at risk of progression and monitor therapeutic intervention and patient management.

## Limitations of the pilot study

The main limitations of the pilot study include (i) the small number of participants in each of the groups, which in turn limited evaluating the reactive aldehydes by gender, (ii) the number of DN urine samples that were available for analysis, and (iii) no staging information was available for the DN patients. However, despite the limitations of this pilot study, the results suggest that further investigation is warranted.

Novel reactive aldehydes, as described in this manuscript, detected in both urine and plasma could be used to stratify risk of progression for patients with T2DM to DN. Identifying patients at risk of progression would significantly reduce morbidity and mortality in this “high-risk” patient population, allowing better use of hospital resources and earlier intervention in patient management.

## Data availability statement

The raw data supporting the conclusions of this article will be made available by the authors, without undue reservation.

## Ethics statement

The studies involving human participants were reviewed and approved by HSC Chelsea/London Ethics Approval Committee. Research governance permission was granted by Northern Health and Social Care Trust. The patients/participants provided their written informed consent to participate in this study.

## Author contributions

DC, SiB, FJ, StB, MK, JL, PF, TM, and MR: concept and study design. CH, JW, and MR: statistical analysis. JW, MK, JL, PF, and MR: manuscript preparation. CH, SiB, FJ, AS, and MR: sample collection and processing. CH, DC, StB, SiB, FJ, AS, JW, PF, TM, and MR: manuscript review. All authors contributed to the article and approved the submitted version.

## References

[1] Röder PV Wu B Liu Y Han W. Pancreatic regulation of glucose homeostasis. *Exp Mol Med.* (2016) 48:e219. 10.1038/EMM.2016.6 26964835 PMC4892884

[2] Lin X Xu Y Pan X Xu J Ding Y Sun X Global, regional, and national burden and trend of diabetes in 195 countries and territories: an analysis from 1990 to 2025. *Sci Rep.* (2020) 10:14790. 10.1038/S41598-020-71908-9 32901098 PMC7478957

[3] International Diabetes Federation. *Diabetes Facts & Figures.* (2022). Available online at: https://www.idf.org/aboutdiabetes/what-is-diabetes/facts-figures.html (accessed June 23, 2022).

[4] The Lancet. Diabetes—a global threat. *Lancet.* (2009) 373:1735. 10.1016/S0140-6736(09)60954-5 19465209

[5] Khan MAB Hashim MJ King JK Govender RD Mustafa H Kaabi JAl. Epidemiology of type 2 diabetes - global burden of disease and forecasted trends. *J Epidemiol Glob Health.* (2020) 10:107–11. 10.2991/JEGH.K.191028.001 32175717 PMC7310804

[6] Williams R Karuranga S Malanda B Saeedi P Basit A Besançon S Global and regional estimates and projections of diabetes-related health expenditure: results from the International Diabetes Federation Diabetes Atlas, 9th edition. *Diabetes Res Clin Pract.* (2020) 162:108072. 10.1016/j.diabres.2020.108072 32061820

[7] Buhary B Almohareb O Aljohani N Alzahrani S Elkaissi S Sherbeeni S Glycemic control and pregnancy outcomes in patients with diabetes in pregnancy: a retrospective study. *Indian J Endocrinol Metab.* (2016) 20:481–90. 10.4103/2230-8210.183478 27366714 PMC4911837

[8] Ghosh S Mahalanobish S Sil PC. Diabetes: discovery of insulin, genetic, epigenetic and viral infection mediated regulation. *Nucleus.* (2021) 1:1–15. 10.1007/S13237-021-00376-X 34629548 PMC8491600

[9] Centers for Disease Control and Prevention[CDC]. *Type 2 Diabetes.* (2022). Available online at: https://www.cdc.gov/diabetes/basics/type2.html (accessed June 23, 2022).

[10] Pitocco D Tesauro M Alessandro R Ghirlanda G Cardillo C. Oxidative stress in diabetes: implications for vascular and other complications. *Int J Mol Sci.* (2013) 14:21525–50. 10.3390/IJMS141121525 24177571 PMC3856020

[11] Yuan CM Nee R Ceckowski KA Knight KR Abbott KC. Diabetic nephropathy as the cause of end-stage kidney disease reported on the medical evidence form CMS2728 at a single center. *Clin Kidney J.* (2017) 10:257. 10.1093/CKJ/SFW112 28396744 PMC5381235

[12] Kidney Research UK. *Kidney Research UK and Diabetes UK Joint Statement.* (2022). Available online at: https://www.kidneyresearchuk.org/research/partnerships-collaboration/kidney-research-uk-and-diabetes-uk-joint-statement/ (accessed July 4, 2022).

[13] Zhou Z Chaudhari P Yang H Fang AP Zhao J Law EH Healthcare resource use, costs, and disease progression associated with diabetic nephropathy in adults with type 2 diabetes: a retrospective observational study. *Diabetes Ther.* (2017) 8:555–71. 10.1007/S13300-017-0256-5 28361464 PMC5446382

[14] Manceur AM Durkin M Kharat A Bookhart B Lafeuille MH Pilon D Costs associated with renal and cardiovascular events among patients with type 2 diabetes mellitus and nephropathy: a cost model based on the CREDENCE clinical trial. *Curr Med Res Opin.* (2020) 36:563–70. 10.1080/03007995.2019.1708285 31916465

[15] Komenda P Ferguson TW Macdonald K Rigatto C Koolage C Sood MM Cost-effectiveness of primary screening for CKD: a systematic review. *Am J Kidney Dis.* (2014) 63:789–97. 10.1053/J.AJKD.2013.12.012 24529536

[16] Manns B Hemmelgarn B Tonelli M Au F Carter Chiasson T Dong J Population based screening for chronic kidney disease: cost effectiveness study. *BMJ.* (2010) 341:1036. 10.1136/BMJ.C5869 21059726 PMC2975430

[17] Tziomalos K Athyros VG. Diabetic nephropathy: new risk factors and improvements in diagnosis. *Rev Diabet Stud.* (2015) 12:110–8. 10.1900/RDS.2015.12.110 26676664 PMC5397986

[18] Ravid M Brosh D Ravid-Safran D Levy Z Rachmani R. Main risk factors for nephropathy in type 2 diabetes mellitus are plasma cholesterol levels, mean blood pressure, and hyperglycemia. *Arch Intern Med.* (1998) 158:998–1004. 10.1001/ARCHINTE.158.9.998 9588433

[19] Duran-Salgado MB Rubio-Guerra AF. Diabetic nephropathy and inflammation. *World J Diabetes.* (2014) 5:393. 10.4239/WJD.V5.I3.393 24936261 PMC4058744

[20] Brosius FC. New insights into the mechanisms of fibrosis and sclerosis in diabetic nephropathy. *Rev Endocr Metab Disord.* (2008) 9:245–54. 10.1007/S11154-008-9100-6 18726161 PMC3776415

[21] Qian Y Feldman E Pennathur S Kretzler M Brosius FC. From fibrosis to sclerosis: mechanisms of glomerulosclerosis in diabetic nephropathy. *Diabetes.* (2008) 57:1439–45. 10.2337/db08-0061 18511444 PMC4239998

[22] Persson F Rossing P. Diagnosis of diabetic kidney disease: state of the art and future perspective. *Kidney Int Suppl.* (2018) 8:2. 10.1016/J.KISU.2017.10.003 30675433 PMC6336222

[23] Lingli X Qing Z Wenfang X. Diagnostic value of the modification of diet in renal disease and chronic kidney disease epidemiology collaboration equations in diabetic patients: a systematic review and meta-analysis. *J Int Med Res.* (2020) 48:0300060520925950. 10.1177/0300060520925950 32589856 PMC7436805

[24] Moore AEB Park-Holohan SJ Blake GM Fogelman I. Conventional measurements of GFR using 51Cr-EDTA overestimate true renal clearance by 10 percent. *Eur J Nucl Med Mol Imaging.* (2003) 30:4–8. 10.1007/S00259-002-1007-Y 12483403

[25] Hurrle S Hsu WH. The etiology of oxidative stress in insulin resistance. *Biomed J.* (2017) 40:257. 10.1016/J.BJ.2017.06.007 29179880 PMC6138814

[26] Tangvarasittichai S. Oxidative stress, insulin resistance, dyslipidemia and type 2 diabetes mellitus. *World J Diabetes.* (2015) 6:456. 10.4239/WJD.V6.I3.456 25897356 PMC4398902

[27] Masschelin PM Cox AR Chernis N Hartig SM. The impact of oxidative stress on adipose tissue energy balance. *Front Physiol.* (2020) 10:1638. 10.3389/FPHYS.2019.01638/BIBTEX PMC6987041 32038305

[28] De Geest B Mishra M. Role of oxidative stress in diabetic cardiomyopathy. *Antioxidants.* (2022) 11:784. 10.3390/ANTIOX11040784 35453469 PMC9030255

[29] Manna P Jain SK. Obesity, oxidative stress, adipose tissue dysfunction, and the associated health risks: causes and therapeutic strategies. *Metab Syndr Relat Disord.* (2015) 13:423. 10.1089/MET.2015.0095 26569333 PMC4808277

[30] Marseglia L Manti S D’Angelo G Nicotera A Parisi E Di Rosa G Oxidative stress in obesity: a critical component in human diseases. *Int J Mol Sci.* (2014) 16:378–400. 10.3390/IJMS16010378 25548896 PMC4307252

[31] Zhou Y Li H Xia N. The interplay between adipose tissue and vasculature: role of oxidative stress in obesity. *Front Cardiovasc Med.* (2021) 8:650214. 10.3389/FCVM.2021.650214 33748199 PMC7969519

[32] Matsuda M Shimomura I. Increased oxidative stress in obesity: implications for metabolic syndrome, diabetes, hypertension, dyslipidemia, atherosclerosis, and cancer. *Obes Res Clin Pract.* (2013) 7:e330–41. 10.1016/J.ORCP.2013.05.004 24455761

[33] Harkin C Smith KW MacKay CL Moore T Brockbank S Ruddock M Spatial localization of β-unsaturated aldehyde markers in murine diabetic kidney tissue by mass spectrometry imaging. *Anal Bioanal Chem.* (2022) 414:6657. 10.1007/S00216-022-04229-7 35881173 PMC9411223

[34] Harkin C Cobice D Brockbank S Bolton S Johnston F Strzelecka A Biomarkers for detecting kidney dysfunction in type-2 diabetics and diabetic nephropathy subjects: a case-control study to identify potential biomarkers of DN to stratify risk of progression in T2D patients. *Front Endocrinol.* (2022) 13:887237. 10.3389/FENDO.2022.887237 35846341 PMC9276980

[35] Bossuyt PM Reitsma JB Bruns DE Gatsonis CA Glasziou PP Irwig L STARD 2015: an updated list of essential items for reporting diagnostic accuracy studies. *BMJ.* (2015) 351:h5527. 10.1136/bmj.h5527 26511519 PMC4623764

[36] R Core Team. *R: A Language and Environment for Statistical Computing.* Vienna: R Foundation Statistical Computing (2022).

[37] Furukawa S Fujita T Shimabukuro M Iwaki M Yamada Y Nakajima Y Increased oxidative stress in obesity and its impact on metabolic syndrome. *J Clin Invest.* (2017) 114:1752–61. 10.1172/JCI21625 15599400 PMC535065

[38] Cho YE Kim HS Lai C Stanfill A Cashion A. Oxidative stress is associated with weight gain in recipients at 12-months following kidney transplantation. *Clin Biochem.* (2016) 49:237–42. 10.1016/J.CLINBIOCHEM.2015.11.002 26545907 PMC4744494

[39] Morrow JD. Is oxidant stress a connection between obesity and atherosclerosis? *Arterioscler Thromb Vasc Biol.* (2003) 23:368–70. 10.1161/01.ATV.0000063107.86298.FD 12639824

[40] Ellulu MS Patimah I Khaza’ai H Rahmat A Abed Y. Obesity and inflammation: the linking mechanism and the complications. *Arch Med Sci.* (2017) 13:851. 10.5114/AOMS.2016.58928 28721154 PMC5507106

[41] Gepstein V Weiss R. Obesity as the main risk factor for metabolic syndrome in children. *Front Endocrinol.* (2019) 10:568. 10.3389/FENDO.2019.00568/BIBTEX PMC6706788 31474943

[42] Singh S Brocker C Koppaka V Chen Y Jackson BC Matsumoto A Aldehyde dehydrogenases in cellular responses to oxidative/electrophilic stress. *Free Radic Biol Med.* (2013) 56:89–101. 10.1016/J.FREERADBIOMED.2012.11.010 23195683 PMC3631350

[43] Jaganjac M Tirosh O Cohen G Sasson S Zarkovic N. Reactive aldehydes–second messengers of free radicals in diabetes mellitus. *Free Radic Res.* (2013) 47(Suppl 1):39–48. 10.3109/10715762.2013.789136 23521622

[44] Catalá A Díaz M. Editorial: impact of lipid peroxidation on the physiology and pathophysiology of cell membranes. *Front Physiol.* (2016) 7:423. 10.3389/FPHYS.2016.00423 27713704 PMC5031777

[45] Ryan MJ Dudash HJ Docherty M Geronilla KB Baker BA Haff GG Vitamin E and C supplementation reduces oxidative stress, improves antioxidant enzymes and positive muscle work in chronically loaded muscles of aged rats. *Exp Gerontol.* (2010) 45:882. 10.1016/J.EXGER.2010.08.002 20705127 PMC3104015

[46] Patlevič P Vašková J Pavol Švorc J Vaško L Švorc P. Reactive oxygen species and antioxidant defense in human gastrointestinal diseases. *Integr Med Res.* (2016) 5:250. 10.1016/J.IMR.2016.07.004 28462126 PMC5390420

[47] Auten RL Davis JM. Oxygen toxicity and reactive oxygen species: the devil is in the details. *Pediatr Res.* (2009) 66:121–7. 10.1203/pdr.0b013e3181a9eafb 19390491

[48] Losada M Alio JL. Malondialdehyde serum concentration in type 1 diabetic with and without retinopathy. *Doc Ophthalmol.* (1996) 93:223–9. 10.1007/BF02569062 9550350

[49] Polak M Zagórski Z. Lipid peroxidation in diabetic retinopathy - PubMed. *Ann Univ Mariae Curie-Sklodowska.* (2004) 59:434–7. 16146026

[50] Dham D Roy B Gowda A Pan G Sridhar A Zeng X 4-Hydroxy-2-nonenal, a lipid peroxidation product, as a biomarker in diabetes and its complications: challenges and opportunities. *Free Radic Res.* (2021) 55:547–61. 10.1080/10715762.2020.1866756 33336611 PMC8260649

[51] Ali TK Matragoon S Pillai BA Liou GI El-Remessy AB. Peroxynitrite mediates retinal neurodegeneration by inhibiting nerve growth factor survival signaling in experimental and human diabetes. *Diabetes.* (2008) 57:889–98. 10.2337/DB07-1669 18285558

[52] Dator RP Solivio MJ Villalta PW Balbo S. Bioanalytical and mass spectrometric methods for aldehyde profiling in biological fluids. *Toxics.* (2019) 7:32. 10.3390/TOXICS7020032 31167424 PMC6630274

[53] Harkin C Smith KW Cruickshank FL Logan Mackay C Flinders B Heeren RMA On-tissue chemical derivatization in mass spectrometry imaging. *Mass Spectrom Rev.* (2021) 41:662–94. 10.1002/MAS.21680 33433028 PMC9545000

[54] Nelson MAM Baba SP Andersonc EJ. Biogenic aldehydes as therapeutic targets for cardiovascular disease. *Curr Opin Pharmacol.* (2017) 33:56–63. 10.1016/J.COPH.2017.04.004 28528297 PMC5563970

[55] Máčová L Bičíková M. Vitamin D: current challenges between the laboratory and clinical practice. *Nutrients.* (2021) 13:1758. 10.3390/nu13061758 34064098 PMC8224373

