# Supplementary material for: Analysis of reactive aldehydes in urine and plasma of type-2 diabetes mellitus patients through liquid chromatography-mass spectrometry: Reactive aldehydes as potential markers of diabetic nephropathy
Source: Front Nutr. 2023 Jan 16;9:997015. doi: 10.3389/fnut.2022.997015 (PMC9885194; doi:10.3389/fnut.2022.997015)
Supplement: Supplementary file 1 [file Data_Sheet_1.docx]

**Supplementary 1**

**Validation results of the LC-MS method for RAs “based on” m10 bioanalytical method validation guidelines.**

**Lower Limit of Quantification (LLOQ)**

The plasma LLOQ for all reactive aldehydes (RAs) was 0.01 nM (precision at 10.1-15.6% and accuracy of 85.2-115.4%, n=6)

**Selectivity**

The selectivity was determined by co-elution assessment analysis of wash, standard 0 (matrix blanks, 0 nM) and (LLOQ 0.5 nM and 1.0 nM). Neither blank nor solvent washes showed peaks corresponding to RAs.

**Matrix effect**

RAs free plasma and tissue was used to matrix match. Peak shape, size and quality showed no significant difference between blank and matrix for all RAs in pooled plasma samples. For spiked samples the matrix effect was determined across two concentration levels (1.0 and 25 nM for plasma) with observed accuracies of 85.5-92.3% (plasma) for all RAs and CV% ranging from 3.4-12.2%. These results show that the matrix effects met the acceptance criteria, and no significant matrix effect was present in the RAs free plasma.

**Calibration and dynamic range**

Calibration curves were obtained from matrix matching calibration standards (STD 1-7), with the lowest concentration standard at the respective LLOQ (Calibration was assessed by evaluating the deviation of standards from the nominal concentration. All standards passed back calculated accuracy at 100±20% with RAs range 88.0-110.0%. A linear regression analysis was performed against ISTD (triplicate) linearity range (0.01-100 nM), linear regression, and squared correlation coefficient r^2^ were within 0.994-0.999. Nominals vs. back calculated concentrations linear regression analysis for all RAs were within 0.994-0.997. No residual trends were observed.

**Accuracy and precision**

The accuracy and precision were determined at 1.0 (LQC) and 25 (HQC) nM. Sample size was n=6 across two separate analytical runs. Intra-day accuracy ranged between 89.43-99.5% and precision did not exceed 3.2% for all RAs. Inter-day accuracy ranged between 93.1%-102.3% and precision did not exceed 6.71%. Intra analyst precision passed at all 3 analysts with lowest 88.5% accuracy and CV% with the highest result of 3.25%. Inter-analyst accuracy passed at 89.7% and CV% passed at 4.8%.

**Carry over**

No signals were observed for RAs in a solvent injection followed by HQCs injection showing no significant carry over.

**Stability**

Autosampler stability was determined for all RAs after sample preparation. Samples were stored at 4˚C for 48 hours after analysis. All results met acceptance criteria after 48 hours for both all RAs with levels against T=0 for all RAs above 85%.
