# Supplementary material for: Analysis of reactive aldehydes in urine and plasma of type-2 diabetes mellitus patients through liquid chromatography-mass spectrometry: Reactive aldehydes as potential markers of diabetic nephropathy
Source: Front Nutr. 2023 Jan 16;9:997015. doi: 10.3389/fnut.2022.997015 (PMC9885194; doi:10.3389/fnut.2022.997015)
Supplement: Supplementary file 2 [file Table_2.DOCX]

**Supplementary 2**

**Supplementary Table 1** Patient Demographics

|  | **Control** | **T2DM** | **DN** | **p value** |
| --- | --- | --- | --- | --- |
| **Factor** | **Mean ± SD** | **Mean ± SD** | **Mean ± SD** | **(KW)** |
| Age | 59.0 ± 13.8 (n=26) | 62.2 ± 9.1 (n=32) | 73.1 ± 9.0 (n=30) | <0.001 |
| Sex (Male) | 6/26 (23.1%) | 20/32 (62.5%) | 18/30 (60%) | 0.005 |
| BMI | 26.3 ± 3.9 (n=25) | 34.4 ± 6.8 (n=16) | 33.8 ± 6.1 (n=10) | <0.001 |
| Alcohol (Never) | 3/26 (11.5%) | 4/32 (12.5%) | 8/30 (26.7%) | 0.224 |
| Smoker (Current) | 0/26 (0%) | 5/32 (15.6%) | 5/30 (16.7%) | 0.003 |
| Smoker (Never) | 19/26 (73.1%) | 16/32 (50%) | 7/30 (23.3%) | 0.003 |
| Smoker (Past) | 7/26 (26.9%) | 11/32 (34.4%) | 18/30 (60%) | 0.003 |
| BP Diastolic (mmHg) | 78.2 ± 10.1 (n=24) | 81.3 ± 8.4 (n=32) | 75.3 ± 11.2 (n=30) | 0.065 |
| BP Systolic (mmHg) | 125.4 ± 13.8 (n=24) | 139.1 ± 19.4 (n=32) | 146.9 ± 23.9 (n=30) | 0.001 |
| Cardiovascular Family History | 11/26 (42.3%) | 14/32 (43.8%) | 12/29 (41.4%) | 0.982 |
| Cardiovascular Comorbidities | 4/26 (15.4%) | 9/32 (28.1%) | 18/30 (60%) | 0.001 |
| DN Family History | 0/26 (0%) | 1/32 (3.1%) | 4/29 (13.8%) | 0.065 |
| T2DM Duration (years) |  | 10.8 ± 5.8 (n=32) | 15.0 ± 6.3 (n=13) | 0.063 |
| T2DM Family History | 7/26(26.9%) | 19/32 (59.4%) | 16/29 (55.2%) | 0.032 |
| Distal renal tubular acidosis | 0/26 (0%) | 0/32 (0%) | 1/30 (3.3%) | 0.376 |
| Kidney Cysts | 1/26 (3.8%) | 0/32 (0%) | 2/30 (6.7%) | 0.348 |
| Kidney Infection | 8/26 (30.8%) | 5/32 (15.6%) | 7/30 (23.3%) | 0.390 |
| Kidney stones | 0/26 (0%) | 4/32 (12.5%) | 6/30 (20%) | 0.061 |
| Neuropathy | 0/26 (0%) | 5/32 (15.6%) | 5/30 (16.7%) | 0.093 |
| Retinopathy | 0/26 (0%) | 6/32 (18.8%) | 14/30 (46.7%) | <0.001 |
| Cancer | 25/26 (96.2%) | 31/32 (96.9%) | 29/30 (96.7%) | 0.558 |

.
